# Unusual grafts for living-donor liver transplantation

**DOI:** 10.1186/s40001-023-01428-5

**Published:** 2023-10-24

**Authors:** Seung Hyuk Yim, Eun-Ki Min, Mun Chae Choi, Deok-Gie Kim, Dai Hoon Han, Dong Jin Joo, Jin Sub Choi, Myong Soo Kim, Gi Hong Choi, Jae Geun Lee

**Affiliations:** Department of Surgery, The Research Institute for Transplantation, Yonsei University College of Medicine, 50-1 Yonsei-ro, Seodaemun-gu, Seoul 03722 South Korea

**Keywords:** Extended left liver plus caudate lobe graft, Right anterior section graft, Right posterior section graft, Donor safety, Surgical outcomes

## Abstract

**Purpose:**

Unusual grafts, including extended left liver plus caudate lobe, right anterior section, and right posterior section grafts, are alternatives to left and right lobe grafts for living-donor liver transplantation. This study aimed to investigate unusual grafts from the perspectives of recipients and donors.

**Methods:**

From 2016 to 2021, 497 patients received living-donor liver transplantation at Severance Hospital. Among them, 10 patients received unusual grafts. Three patients received extended left liver plus caudate lobe grafts, two patients received right anterior section grafts, and five patients received right posterior section grafts. Liver volumetrics and anatomy were analyzed for all recipients and donors. We collected data on laboratory examinations (alanine aminotransferase, total bilirubin, international normalized ratio), imaging studies, graft survival, and complications. A 1:2 ratio propensity-score matching method was used to reduce selection bias and balance variables between the unusual and conventional graft groups.

**Results:**

The median of Model for End-stage Liver Disease score of unusual graft recipients was 13.5 (interquartile range 11.5–19.3) and that of graft–recipient weight ratio was 0.767 (0.7–0.9). ABO incompatibility was observed in four cases. The alanine aminotransferase level, total bilirubin level, and international normalized ratio decreased in both recipients and donors. Unusual and conventional grafts had similar survival rates (*p* = 0.492). The right and left subgroups did not differ from each counter-conventional subgroup (*p* = 0.339 and *p* = 0.695, respectively). The incidence of major complications was not significantly different between unusual and conventional graft recipients (*p* = 0.513). Wound seromas were reported by unusual graft donors; the complication ratio was similar to that in conventional graft donors (*p* = 0.169).

**Conclusion:**

Although unusual grafts require a complex indication, they may show feasible surgical outcomes for recipients with an acceptable donor complication.

## Introduction

Liver transplantation (LT) is becoming a standard treatment for patients with liver cirrhosis. However, it is challenging to overcome the problem of deceased donor shortage, especially in Far East Asian countries where the number of deceased donors is lower than that in western countries [1]. The concept of living-donor LT (LDLT) emerged in 1994 to address this issue. The initial graft of choice for LDLT was the left lobe of the liver, but the volume of the graft was insufficient to avoid small-for-size-graft syndrome. Thus, the right lobe emerged as an alternative option. However, the large volume of the right lobe leads to donor safety issues [2]. To secure residual liver volume of > 30% of the standard liver volume [3], right anterior section [4, 5], right posterior section [6], and extended left liver plus caudate lobe grafts [7] were introduced. The right anterior section graft is a partial liver graft of Couinaud’s segments 5 and 8 with the middle hepatic vein (HV). The right posterior section graft is composed of segments 6 and 7 with the right HV. The extended left liver plus caudate lobe graft is composed of segments 1, 2, 3, and 4 with the left HV and caudate vein. These grafts ensure the acquisition of grafts larger than left lobe grafts and are associated with improved donor safety compared to that with right lobe grafts. Because of the complex indication of the grafts due to the variances in liver anatomy and a challenging transection plane, it is rarely used. However, as a high-volume LDLT center, our institution has employed unusual grafts for donors who were unsuitable for conventional grafts. Experienced transplant surgeons have been performing accurate donor hepatectomy and recipient management for unusual grafts [5]. The outcome of unusual grafts has not been reported before, because as the name alone suggests, these grafts are rarely used. Therefore, in this study, we present the outcomes of LDLT with the unusual grafts from the perspective of recipients and donors.

## Patients and methods

### Patients

**Fig. 1:**
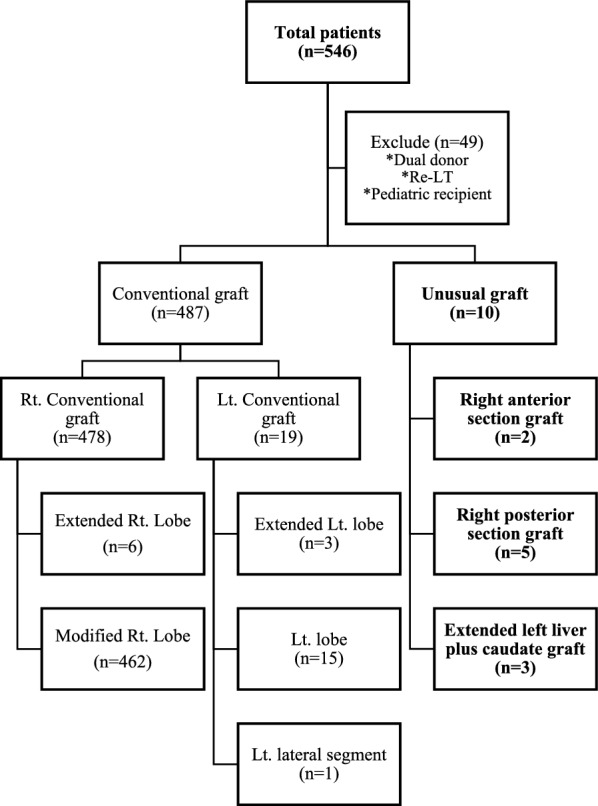
Study flow diagram. DDLT, deceased donor liver transplantation; re-LT, second liver transplantation; Lt: left; Rt: right

Data of 497 patients who underwent LDLT at Severance Hospital from January 2016 to December 2021 were retrospectively collected. Pediatric patients and patients who received grafts from two separate donors or underwent re-liver transplantation were excluded. The donors of each recipient were paired during data collection. Ten patients received unusual grafts (Fig. 1). All recipients and donors underwent a series of evaluations to investigate liver anatomy, including dynamic computed tomography, magnetic resonance imaging including cholangiopancreatography, and liver fibrosis scanning.

### Graft selection

The graft volumes were estimated using computed tomography volume analysis. Initially, the volumes of conventional liver grafts, such as the extended right lobe, modified right lobe, or extended left lobe, were calculated based on the residual liver volumes and the graft–recipient weight ratio (GRWR). The residual liver volume needed to exceed 30%, and the GRWR had to be greater than 0.8 for these calculations. In cases where the donors were not suitable for the conventional grafts, and there were multiple eligible donors for the recipient, we selected the preferred donor who could provide a conventional graft. However, when the recipient had only one potential donor, we considered the option of utilizing the unusual grafts. We assessed the anatomical variances in the portal vein (PV), hepatic artery (HA), and hepatic duct (HD) of the donor. Many types of HA branching have been reported, from the standard anatomy to the left HA arising from the left gastric artery and the right HA arising from the superior mesenteric artery [8]. Additionally, there is a variant in which the segment 4 artery branches out from the right HA. PVs are usually of three different types. Type 1 PV variant is the standard type, which shows the bifurcation of the right and left PVs from the main PV. Type 2 PV variant is the trifurcation type, in which the main PV branches into the right anterior, right posterior, and left PVs. Type 3 PV variant shows separate right posterior PV from the main PV and bifurcation of the right anterior and left PVs [9]. The intra-HD also has many variations [10]. The relation between the HA, PV, and HD of each section is also a major factor for consideration.

Considering anatomical variances and GRWR, the donor who was unsuitable for donating conventional grafts was investigated for their eligibility to be the donor of unusual graft. Based on their anatomical variances, possible unusual graft was assessed. Each unusual graft has its favorable anatomy, and it will be described in detail later. We visualized each unusual graft using Synapse 3D Liver Analysis (FUJIFILM Medical Systems U.S.A. Inc., Valhalla, NY, USA). The detailed information about graft selection is shown in Table 1.

**Table 1:** Graft selection data

	Donor 1	Donor 2	Donor 3	Donor 4	Donor 5^†^	Donor 6	Donor 7^‡^	Donor 8^*^	Donor 9	Donor 10
Total liver volume, ml	1538	1823	1260	2015	1111	1721	1560	1425	2808	1265
*Modified right lobe*										
Graft volume, ml	1102.0	1381.0	960	1425.0	798.0	1249.0	988.4	1080.0	2075.0	890.0
Residual volume, %	28.3	24.2	23.8	29.3	28.2	27.4	36.6	24.2	26.1	29.6
GRWR	1.6	1.6	1.2	2.0	1.9	2.4	1.5	1.5	3.0	1.6
*Extended left liver plus caudate lobe*										
Graft volume, ml	436.0	442.0	300	**590.0**	313.0	**472.0**	**571.6**	345.0	733.0	375.0
Residual volume, %	71.7	75.8	71.8	**70.7**	71.8	**72.6**	**63.4**	75.8	73.9	70.4
*GRWR*	0.6	0.5	0.38	**0.8**	0.7	**0.9**	**0.9**	0.5	1.1	0.7
*Right posterior section*										
Graft volume, ml	**734.0**	**678.0**	304.0	430.0	357.0	636.0	480.0	**523.1**	**910.6**	**551.0**
Residual volume, %	**52.3**	**62.8**	75.8	78.7	67.9	63	69.2	**63.3**	**67.6**	**56.4**
GRWR	**1.0**	**0.8**	0.42	0.6	0.9	1.2	0.7	**0.8**	**1.3**	**1.0**
*Right anterior section*										
Graft volume, ml	368.0	703.0	**655.0**	995.0	**441.0**	613.0	508.4	556.9	1164.4	339.0
Residual volume, %	76.1	61.4	**48.0**	50.6	**60.3**	64.4	67.4	60.9	58.5	73.2
GRWR	0.5	0.8	**0.83**	1.4	**1.1**	1.2	0.8	0.8	1.7	0.6
*Anatomical variance*										
Portal vein	Type 3	Type 3	Type 3	Type 1	Type 1	Type 1	Type 2	Type 2	Type 1	Type 3
Hepatic artery		Extrahepatic bifurcation of right HA	Extrahepatic bifurcation of right HA	Early bifurcation of left HA	Extrahepatic bifurcation of right HA	Early bifurcation of left HA	Early bifurcation of left HA	Extrahepatic bifurcation of right HA	**A4 from right HA**	Extrahepatic bifurcation of right HA
Bile duct										Right posterior duct drains into left duct

GRWR: graft–recipient weight ratio; HA: hepatic artery

^†^Graft had short stump of Rt. posterior HA

^‡^Donor had weight reduction, liver fibroscan: 305db/m (S3) → 281db/m (S2)

^*^Donor had weight reduction, liver fibroscan: 312db/m (S3) → 307db/m (S2)

### Right anterior section graft (Fig. 2A)

**Fig. 2:**
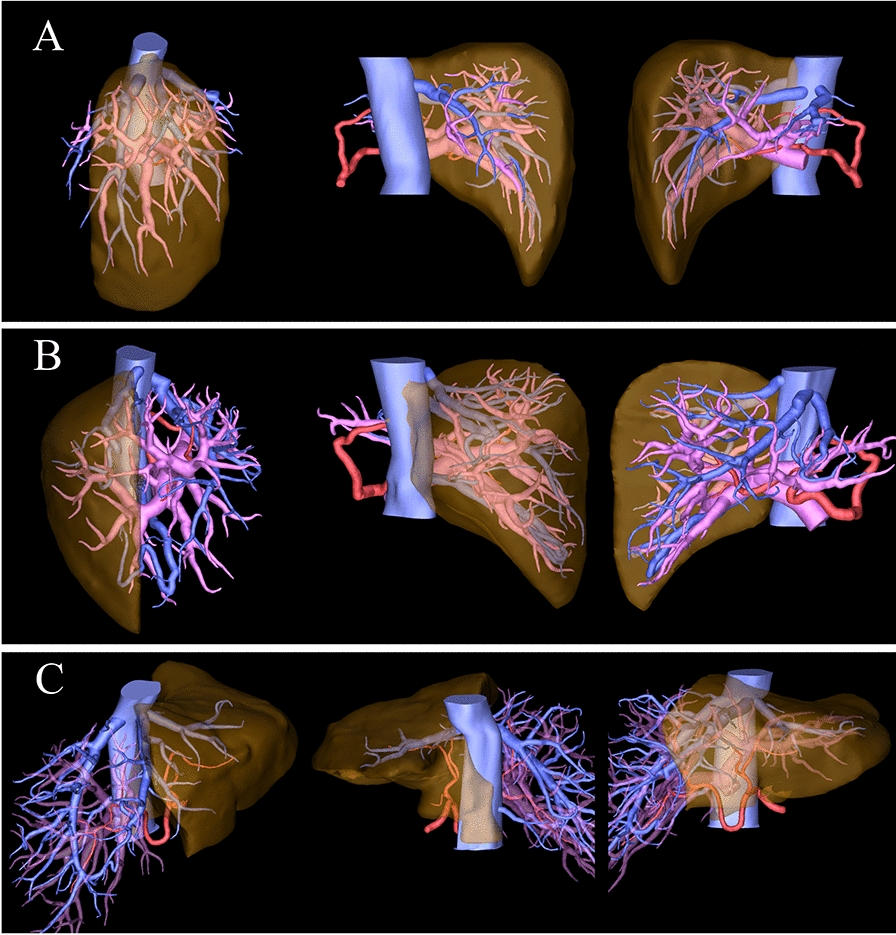
Unusual grafts. A Right anterior section graft. B Right posterior section graft. C Extended left liver plus caudate lobe graft

#### Suitable anatomy

The extrahepatic second-order bifurcation of the right HA favors right anterior section graft procurement because it can ensure a longer stump of the right anterior HA [4]. No segment 4 artery branching from the right HA is also helpful. Type 3 PV variant, in which the posterior PV is separate from the main PV, is the favorable choice for right anterior section grafts. Separate bile ducts from the anterior and posterior sections are also important. For donors who have dominant volumes of the anterior section compared to those of the posterior section, the right anterior section grafts are favored over other unusual grafts.

#### Donor graft sectionectomy

After ligamentum teres, falciform, coronary, and right triangular ligament divisions, hilar dissection was initiated posterolaterally. The right HA, right PV, and right HD were identified and isolated. The anterior branches of the right HA and PV were identified and isolated separately. The right anterior HA and right anterior PV were clamped to draw a demarcation line with electrocautery. Intraoperative ultrasonography was performed to identify the location of the right HV and middle HV. After confirming the dissection plane, the Cavitron Ultrasonic Surgical Aspirator was used to transect the liver. While parenchymal dissection along the right HV, branches of segments 5 and 8 connected to right HV were ligated gently and divided for reconstruction. Another transection was performed along the middle HV. The right anterior HD was identified and isolated using the Glissonian approach and divided, leaving a 3-mm HD stump for reconstruction. The right anterior HA and right anterior PV were divided gently and the middle HV was sealed with an endovascular stapler and transected. Intraoperative cholangiography was performed to confirm the integrity of the right posterior HD. The graft was flushed with a histidine–tryptophan–ketoglutarate solution. The right HV branches of segments 5 and 8 was reconstructed using the vascular interposition graft (reconstruction of right HV). The proximal end of the interposition graft was anastomosed to the middle HV of the graft for a common channel outflow.

#### Recipient surgery

The stump of the recipient middle HV was left to reconstruct anastomosis. A bigger orifice was created in the middle HV using venotomy to ensure adequate venous outflow. The conjoined graft HV and enlarged recipient middle HV were anastomosed. The recipient right PV was connected to the donor right anterior PV, and the recipient right HA was connected to the donor right anterior HA. Intraoperative duplex ultrasonography was performed to confirm a successful flow of PV, HA and HV. Finally, the recipient right HD and the donor right anterior HD were anastomosed using the duct-to-duct method.

### Right posterior section graft (Fig. 2B)

#### Suitable anatomy

The extrahepatic second-order bifurcation of right HA also favors right posterior section graft procurement. The type 3 PV variant is the ideal choice for right posterior section grafts. Another favorable anatomy is an extrahepatic right posterior HD that drains into the common HD. Additionally, the right posterior HD that runs through the ventral side of the right posterior PV is the most suitable anatomy [11, 12].

#### Donor graft sectionectomy

Division of ligamentum teres to hilar dissection was performed in the same manner as that for right anterior section grafts. The posterior branches of the right HA and PV were identified and isolated separately. The right posterior HA and right posterior PV were clamped to establish a demarcation line and line was drawn with electrocautery. The right HV was identified using intraoperative ultrasonography and dissection plane was established. The Glissonian method was used to identify and isolate the right posterior HD, and parenchymal transection was performed toward the root of the right posterior HD with using the Cavitron Ultrasonic Surgical Aspirator. The right posterior HA and right posterior PV were separated gently and the right HV was transected after sealing with a vascular stapler. Intraoperative cholangiography was performed to confirm the integrity of the right anterior HD.

#### Recipient surgery

The right HV of the donor and the right HV of the recipient were anastomosed. The donor right posterior PV was connected to the recipient right PV, and the donor right posterior HA was connected to the recipient right HA. A successful flow was confirmed using intraoperative duplex ultrasonography. Finally, the duct-to-duct approach was used to anastomose the donor right posterior HD with the recipient right HD. In some cases, the hepaticojejunostomy was utilized to rebuild the bile duct anastomosis.

### Extended left liver plus caudate lobe graft (Fig. 2C)

#### Suitable anatomy

The favorable anatomy for extended left liver plus caudate lobe grafts is the caudate vein draining into the inferior vena cava far from the orifices of the middle and left HVs because it is difficult to anastomose the caudate vein when it is situated near the root of the middle HV or left HV.

#### Donor graft lobectomy

The left HA was isolated and looped. The left PV was isolated at the bifurcation with the Glissonian method, while its transverse portion was left undissected to preserve the caudate lobe branches. Caution should be taken to avoid damage when securing the caudate vein. The left caval ligament was dissected, then the caudate lobe was mobilized from the vena cava while preserving the caudate vein. The transection line was made from the midpoint between the trunks of the right and middle HVs to the right margin of the retrohepatic inferior vena cava. Identified left HA and left PV was clamped to draw demarcation line. The Cavitron Ultrasonic Surgical Aspirator was used to transect the liver, and the left HD was ligated. The left HA and left PV were ligated and the common trunk of middle and left HVs were transected after sealing with a vascular stapler. Caudate veins were separately ligated. Intraoperative cholangiography was performed to confirm the integrity of the right HD.

#### Recipient surgery

A venotomy of the recipient middle HV and left HV were performed to construct a common channel. A venotomy using an end-to-side method was performed on the vena cava to anastomose the caudate vein [13]. End-to-end anastomoses were performed between the recipient conjoined common trunk (middle HV and left HV) and graft conjoined middle-left HV. End-to-end anastomosis was performed with the donor left PV to the recipient left PV and the donor left HA to the recipient left HA. However, in Case 7, the left HA was anastomosed to the recipient gastroduodenal artery due to the dissection of the recipient HA lumen. In general, duct-to-duct anastomosis was favored for bile duct reconstruction, however, in one case (Case 6), hepaticojejunostomy was performed.

### Postoperative outcomes

We collected data on laboratory findings, computed tomography findings, graft survival, overall survival, and complications of recipients and donors. We graded surgical complications using the Clavien–Dindo classification [14], and we classified Grade III or above as major complications. In addition, complications were categorized into the complication of HA, HV (including inferior vena cava), PV, bile duct, and others.

### Statistical analysis

Data are presented as numbers (proportions) for categorical variables and means and standard deviations for continuous variables. Due to the small sample size, non-parametric tests were required. To determine the significance of intergroup differences, Fisher’s exact test was used for categorical variables and the Mann–Whitney U test was used for continuous variables; data are summarized and reported as medians (interquartile ranges). Survival rates were evaluated using the Kaplan–Meier method, and the log-rank test was used for between-group comparisons.

Propensity score matching (PSM) was performed to minimize selection bias and balance variables between the unusual graft and the conventional graft groups. Unusual graft recipients were matched with conventional graft-recipients in a ratio of 1:2 using the nearest neighbor matching algorithm without replacement with distances determined by logistic regression. The caliper was not applied because the unusual graft group only had 10 patients. PSM was performed based on the following variables: recipient model for end-stage liver disease (MELD) score, Milan’s criteria, and donor age, height, and weight.

Statistical analyses were performed using IBM SPSS 26 (SPSS Inc., Chicago, IL) and R 3.5.3 software (R Foundation for Statistical Computing, Vienna, Austria). *p*-values < 0.05 were considered statistically significant.

## Results

### Patients and grafts

Among the 10 unusual graft recipients, five received right posterior section grafts, two received right anterior section grafts, and three received extended left liver plus caudate lobe grafts (Table 2). The median follow-up period was 31.5 months (range, 23 days [recipient death] to 68 months). Six patients had hepatitis B virus hepatocellular carcinoma, and the remaining each had hepatitis C virus hepatocellular carcinoma, alcoholic liver cirrhosis, hepatitis B virus liver cirrhosis, or autoimmune hepatitis. The median MELD score was 13.5 (interquartile range (IQR) 11.5–19.3) and that of GRWR was 0.767 (IQR 0.7–0.9). PSM with a 1:2 ratio resulted in 20 matched pairs for further analysis (Table 3). In the unmatched analysis, the number of patients above Milan’s criteria was significantly different (*p* = 0.049). Further, the donor sex (*p* = 0.006) and weight (*p* = 0.012) were different. After PSM using nearest neighbor matching, both groups were well balanced in all baseline clinicopathological characteristics.

**Table 2:** Characteristics of the recipients of unusual graft and their donors

	Case 1	Case 2	Case 3	Case 4	Case 5	Case 6	Case 7	Case 8	Case 9	Case 10
*Recipient*										
Sex	Male	Male	Male	Male	Female	Female	Female	Male	Male	Male
Age	61	48	56	51	13	71	58	58	65	62
Height, cm	161	173	174	172	146	159	143	173	166.7	164
Weight, kg	68	84.7	76	71	42	51	65	70	69.4	57
BMI	26.23	28.3	25.1	24	19.7	20.17	31.79	23.39	24.97	21.18
Etiology	HBV HCC	HBV HCC	Alcohol	HBV	AIH	HBV HCC	HBV HCC	HBV HCC	HCV Alcohol HCC	HBV HCC
MELD score	17	14	20	32	13	11	8.85	11	13	9
ABO type	O	O	O	A	A	A	A	B	B	AB
Operation time, min	812	639	497	650	625	590	1134	514	456	411
Estimated blood loss, mL	2100	8150	4200	7300	3100	5800	15,500	6900	4000	5000
Postoperative hospital stay, days	35	22	15	36	28	23	12†	35	23	19
Postoperative Outcomes										
Primary non-function	No	No	No	No	No	No	No	No	No	No
Small-for-size syndrome	No	No	No	No	No	No	Yes	No	No	No
HA complication	No	No	No	No	Yes	No	No	No	No	No
Biliary complication	No	Yes	No	Yes	Yes	No	No	Yes	No	No
180-day mortality	No	No	No	No	No	No	Yes	No	No	No
*Donor*										
Sex	Male	Male	Male	Male	Male	Male	Male	Male	Male	Male
Age	34	44	26	48	45	42	52	39	33	33
Height, cm	171	186	177	175	170	165	158	175	180	178
Weight, kg	69	85	83	82	68	55	76	76	87	71.3
BMI	23.6	24.57	26.49	26.77	23.52	20.2	30.44	24.82	26.85	22.5
ABO type	O	B	O	O	B	AB	O	A	B	B
Operation time, min	352	283	385	535	285	408	464	313	271	265
Estimated blood loss, mL	300	450	150	250	100	800	300	600	300	300
Postoperative hospital stay, days	14	7	8	8	9	8	7	8	7	8
Estimated remnant liver volume, %	49.3	61.03	47.9	67.27	67.38	70.79	61.02	54.8	51.3	55.9
*Graft*										
Type, selected	**RPG**	**RPG**	**RAG**	**Ext LLC**	**RAG**	**Ext LLC**	**Ext LLC**	**RPG**	**RPG**	**RPG**
Estimated graft volume, mL	734	678	655.8	590	357	472	571.6	523.1	910.6	551.0
Weight, g	531	639	612	519	410	592	351	452	694	334
Estimated GRWR	1.03	0.80	0.83	0.83	0.85	0.92	0.88	0.75	1.30	0.98
GRWR	0.78	0.75	0.80	0.73	0.97	1.16	0.54	0.64	1.00	0.60

^†^Hospital stay of Case 7 is limited to patient’s second liver transplantation

AIH: autoimmune hepatitis; Ext. LLC: extended left liver plus caudate lobe graft; GRWR: graft–recipient weight ratio; HA: hepatic artery; HBV: B-viral hepatitis; HCC: hepatocellular carcinoma; RAG: right anterior section graft; RPG: right posterior section graft; SFSS: small-for-size syndrome

**Table 3:** Patient demographics

	Before propensity-score matching			After propensity-score matching (all)			After propensity-score matching (right)			After propensity-score matching (left)		
	Conventional (*n* = 487)	Unusual (*n* = 10)	*p*-value	Conventional (*n* = 20)	Unusual (*n* = 10)	*p*-value	Rt. conventional (*n* = 14)	RAG, RPG (*n* = 7)	*p*-value	Lt. conventional (*n* = 6)	Ext. LLC (*n* = 3)	*p*-value
*Recipient*												
Sex: female	152(31.2)	3(30)	1.000	4(20)	3(30)	0.657	4(28.6)	1(14.3)	0.624	2(33.3)	2(66.7)	0.524
Age	56.00 (50.00–61.00)	57.00 (50.25–61.75)	0.751	61.00 (52.25–63.75)	57.00 (50.25–61.75)	0.373	59.50 (56.75–65.00)	56.00 (458.00–61.00)	0.149	55.00 (46.50–63.75)	58.00 (51.00–71.00)	0.548
Height, cm	167.00 (160.00–171.30)	164.50 (155.75–173.25)	0.711	169.00 (165.75–173.50)	164.50 (155.75–173.25)	0.448	163.00 (158.50–170.00)	166.00 (161.000–174.00)	0.400	167.15 (155.90–172.00)	159.00 (143.00–172.00)	0.714
Weight, kg	67.00 (58.00–74.00)	69.00 (55.50–72.25)	0.949	70.50 (62.00–77.00)	69.00 (55.50–72.25)	0.286	68.00 (58.75–75.00)	70.00 (57.00–76.00)	0.913	60.50 (44.75–64.25)	65.00 (51.00–71.00)	0.262
BMI	23.81 (21.97–26.16)	24.55 (21.13–26.28)	0.849	24.31 (21.72–25.73)	24.55 (21.13–26.28)	0.948	24.47 (23.29–26.29)	25.10 (21.45–26.23)	0.585	21.80 (15.94–23.02)	24.00 (20.17–31.79)	0.381
MELD score	13.00 (10.00–19.00)	13.00 (11.00–23.00)	0.464	13.00 (11.00–19.50)	13.00 (11.00–23.00)	0.948	12.50 (8.75–22.00)	13.00 (11.00–17.00)	0.856	25.50 (16.75–31.50)	32.00 (11.00–40.00)	0.714
HTN	113(23.2)	1(10)	0.467	6(30)	1(10)	0.372	4(28.6)	0(0)	0.255	1(16.7)	1(33.3)	1.000
DM	127(26.1)	1(10)	0.465	3(15)	1(10)	1.000	3(21.4)	0(0)	0.521	3(50)	1(33.3)	1.000
HBV	243(49.9)	7(70)	0.339	14(70)	7(70)	1.000	8(57.1)	4(57.1)	1.000	4(66.7)	3(100)	0.500
HCV	27(5.5)	2(20)	0.111	1(5)	2(20)	0.251	1(7.1)	1(14.3)	1.000	0(0)	1(33.3)	0.333
HCC	258(53.0)	7(70)	0.350	17(85)	7(70)	0.372	10(71.4)	5(71.4)	1.000	3(50)	2(66.7)	1.000
MILAN			0.049			0.462			0.376			1.000
Within Milan	176(36.1)	2(20)		7(35)	2(20)		6(42.9)	1(14.3)		2(33.3)	1(33.3)	
Above Milan	82(16.8)	5(50)		10(50)	5(50)		4(28.6)	4(57.1)		1(16.7)	1(33.3)	
*Donor*												
Sex: female	207(42.5)	0(0)	0.006	4(20)	0(0)	0.272	1(7.1)	0(0)	1.000	1(16.7)	0(0)	1.000
Age	34.00 (25.00–42.00)	38.50 (33.00–45.75)	0.127	40.00 (32.50–48.75)	38.50 (33.00–45.75)	0.746	31.50 (24.50–44.25)	34.00 (33.00–44.00)	0.443	39.00 (32.00–50.75)	48.00 (42.00–52.00)	0.381
Height, cm	170.00 (162.00–175.00)	175.00 (168.75–178.50)	0.072	172.00 (167.25–176.00)	175.00 (168.75–178.50)	0.475	178.00 (173.75–184.50)	177.00 (170.00–180.00)	0.856	169.50 (164.25–175.00)	165.00 (158.00–175.00)	0.548
Weight, kg	65.00 (58.50–75.00)	75.50 (68.75–83.50)	0.012	70.00 (62.25–82.88)	75.50 (68.75–83.50)	0.397	75.50 (65.75–87.75)	75.00 (69.00–85.00)	0.585	78.50 (66.65–86.25)	76.00 (55.00–82.00)	0.548
BMI	23.31 (21.36–25.23)	24.53 (23.25–26.79)	0.088	23.75 (22.42–25.66)	24.53 (23.25–26.79)	0.397	23.33 (21.94–26.03)	24.49 (23.53–26.49)	0.400	21.80 (15.94–23.02)	26.78 (20.20–30.44)	1.000
HTN	11(2.3)	0(0)	1.000	0(0)	0(0)	–	1(7.1)	0(0)	1.000	1(16.7)	0(0)	1.000
DM	1(0.2)	0(0)	1.000	0(0)	0(0)	–	1(7.1)	0(0)	1.000	0(0)	0(0)	–

Values are presented as median (interquartile), or number (%) unless otherwise indicated

BMI: body mass index; D: donor; DM: diabetes mellitus; Ext. LLC: extended left liver plus caudate lobe graft; HBV: B-viral hepatitis; HCC: hepatocellular carcinoma; HCV: C-viral hepatitis; HTN: hypertension; MELD: Model for End-stage Liver Disease; R: recipient; RAG: right anterior section graft; RPG: right posterior section graft

### Postoperative outcomes

#### Immediate outcomes

**Fig. 3:**
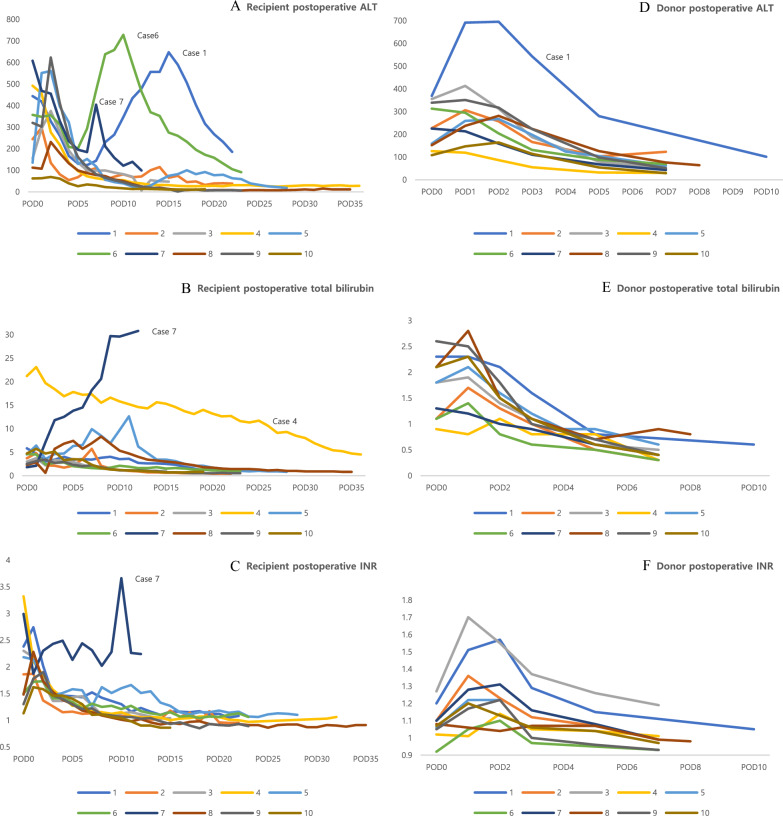
Postoperative laboratory findings. **A** Recipient ALT. **B** Recipient total bilirubin. **C** Recipient INR. **D** Donor ALT. **E** Donor total bilirubin. **F** Donor INR. ALT: alanine aminotransferase; INR: international normalized ratio; POD: postoperative day

Alanine aminotransferase (ALT), total bilirubin, and international normalized ratio (INR) values were evaluated. The follow-up period was confined to the initial hospital stay. The recipient’s ALT, total bilirubin, and INR values showed general decrement. The postoperative laboratory outcomes of the donors showed a similar tendency (Fig. 3).

#### Survival (graft and patient survival)

**Fig. 4:**
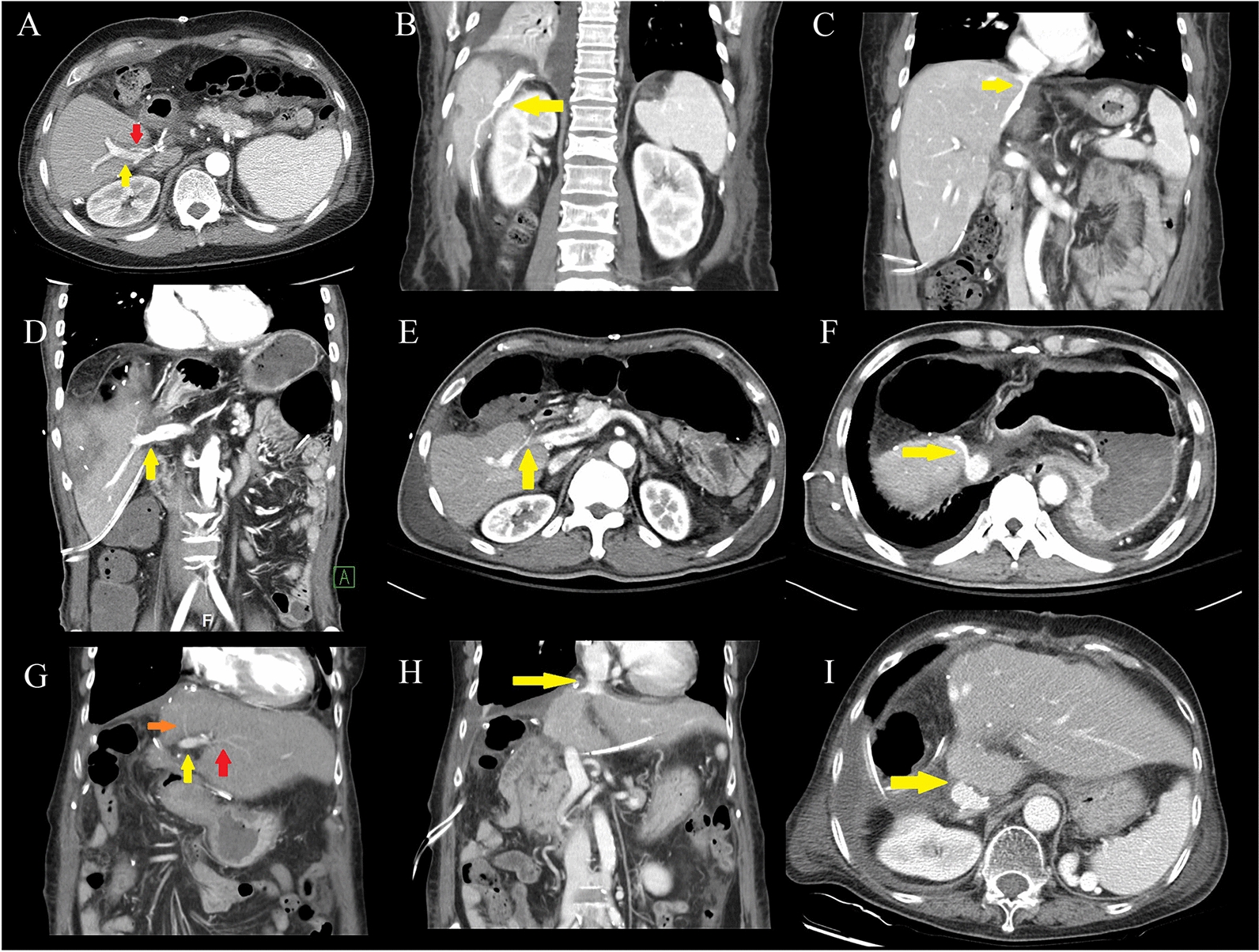
Postoperative imaging studies of right anterior section graft (**A**–**C**), right posterior section graft (**D**–**F**), and extended left liver plus caudate lobe graft (**G**–**I**). **A** Intact flow of PV (yellow) and HA (red) are seen. **B** Reconstructed tributaries of the RHV from segment 5. **C** Anastomosis of the common channel formed by the RHV graft and the middle HV. **D** Anastomosis of the donor right posterior PV to recipient right PV. **E** Intact HA flow. **F** Anastomosis of the RHV to the recipient RHV. **G** Intact flow of left PV (yellow), medial segmental branch of left HA (orange), and lateral segmental branch of left HA (red). (H) Anastomosis of the donor conjoined HV (middle HV, left HV) and recipient conjoined HV. **I** Anastomosis of the caudate vein to the recipient vena cava. PV: portal vein; HV: hepatic vein; HA: hepatic artery; RHV: right hepatic vein

All 479 recipients were divided into the conventional graft recipient and unusual graft recipient groups. The graft survival is shown in Fig. 4. There were no significant differences between the groups before PSM (*p* = 0.778) and after PSM (*p* = 0.492). The same result was observed for patient overall survival (data not shown). Additionally, we performed subgroup analysis of the unusual graft recipient group. Recipients of the right anterior section and right posterior section grafts did not show different graft survival and patient overall survival outcomes than those of right conventional grafts. Moreover, the survival rates of the extended left liver plus caudate lobe graft recipient group were not different from those of the left conventional graft recipient group.

#### Complications

Postoperative complications in liver transplant recipients with unusual grafts and others are summarized in Table 4. The overall complication rate in unusual graft recipients was 60%, while that in conventional graft recipients was 54.8% (*p* = 1.00). Regarding major complications, which were defined as Clavien–Dindo grade III or more, unusual graft recipients had an incidence rate of 50%, while conventional graft recipients had an incidence rate of 37.4% (*p* = 0.51). The types of complications were also investigated. Biliary complications were the most common major complication; 40% unusual graft recipients experienced bile duct problems, while 26.9% conventional graft recipients showed biliary complications (*p* = 0.47). HA and PV complications occurred in one case each. Subgroup analysis was performed in the same manner as the survival rate analysis. The complication rate in right unusual graft and left unusual graft recipients was not significantly different compared to that of unusual graft recipients.

**Table 4:** Complications according to graft types among 497 LDLT

	Right conventional (*n* = 468)	RAG (*n* = 2)	RPG (*n* = 5)	Left conventional (*n* = 19)	Ext. LLC (*n* = 3)	*p*-value^†^	*p*-value^‡^	*p*-value*
*Recipient complication*						**0.834**	**0.38**	**0.33**
Grade 0	232(49.5)	1(50)	2(40)	9(47.3)	1(33.3)			
Grade I	11(2.3)	0(0)	0(0)	0(0)	0(0)			
Grade II	54(11.5)	0(0)	1(20)	3(15.7)	0(0)			
Grade IIIA	135(28.8)	1(50)	2(40)	6(31.5)	1(33.3)			
Grade IIIB	30(6.4)	0(0)	0(0)	1(5.2)	0(0)			
Grade IVA	0(0)	0(0)	0(0)	0(0)	0(0)			
Grade IVB	0(0)	0(0)	0(0)	0(0)	0(0)			
Grade V	6(1.2)	0(0)	0(0)	0(0)	1(33.3)			
Overall complication	254(54.2)	1(50)	3(60)	13(68.4)	2(66.6)	**1.000**	**1.000**	**1.000**
Major complication, grade ≥ 3	173(36.9)	1(50)	2(40)	9(47.3)	2(66.6)	**0.714**	**1.000**	**0.513**
*Types of complication*								
Hepatic artery	21(4.4)	1(50)	0(0)	1(5.2)	0(0)	**0.284**	**1.000**	**0.380**
Hepatic vein	29(6.1)	0(0)	0(0)	0(0)	0(0)	**1.000**		**1.000**
Portal vein	14(2.9)	0(0)	0(0)	0(0)	1(33.3)		**0.136**	**0.266**
Bile duct	127(27.1)	1(50)	2(40)	4(21.0)	1(33.3)	**0.398**	**1.00**	**0.471**
*Donor complication*								**0.169**
Grade 0	460(98.3)	2(100)	4(80)	19(100)	3(100)			
Grade I	2(0.4)	0(0)	0(0)	0(0)	0(0)			
Grade II	3(0.6)	0(0)	1(20)	0(0)	0(0)			
Grade IIIA	3(0.6)	0(0)	0(0)	0(0)	0(0)			

Data are given as number (%)

The conventional group was composed of the right lobe, extended right lobe, left lobe, extended left lobe, and left lateral segment grafts. The unusual group was composed of the right anterior section, right posterior section, or extended left liver plus caudate lobe

^†^*p* values were calculated according to graft types of right lobe (Rt. conventional vs. RAG + RPG) using Fisher’s exact test

^‡^*p* values were calculated according to graft types of left lobe (Lt. conventional vs. Ext. LLC) using Fisher’s exact test

^*^p values of the conventional graft versus the unusual graft comparisons were calculated using Fisher’s exact test

Ext. LLC, extended left liver plus caudate lobe graft; RAG, right anterior section graft; RPG, right posterior section graft

Regarding donor outcomes, only one case (10%) from the unusual graft group showed complications—a wound seroma that occurred 9 days after surgery. Eight donors (1.6%) from the conventional graft group experienced complications (*p* = 1.69).

## Discussion

Because of the shortage of the deceased donor pool, LDLT has become popular in Asian countries. Unlike deceased donor liver transplantation, LDLT has donor safety issues because the residual liver volume should be at least > 30% of the standard liver volume. However, to avoid small-for-size syndrome in the recipient, the graft should be > 0.8% of the recipient’s weight. Thus, the optimal graft should ensure enough volume of the liver for both the donor and the recipient. However, in certain cases, conventional grafts may be unsuitable for either the recipient or the donor. To address this issue, Miyagawa et al. suggested extended left liver plus caudate lobe as an LDLT graft to extend the donor pool  [15]. Moreover, Sugawara et al. introduced the right posterior section graft and Suh et al. described their experience with using the right anterior section as an LDLT graft [4, 6]. Because each donor has diverse volume distribution of each segment, the volume of each segment must be measured separately. For example, when the donor has a considerable right anterior section volume, the right anterior section graft should be considered first; when the donor has considerable posterior section volume, the right posterior section graft should be considered; when the donor has a large caudate lobe, the extended left liver plus caudate lobe graft should be considered.

However many previous studies have emphasized not to use unusual grafts based on mere volume estimates [4, 16–18]. There are many factors to consider, including the donor’s age, the existence of fatty liver, the method for volume evaluation, and so on [19, 20]. Another important factor for consideration is the donor’s anatomical variance. Each graft has a favorable anatomical structure variant. The extrahepatic bifurcation of the second-order branches of the right HA ensures longer HA roots for both the right anterior section and right posterior section grafts. Type 3 variant PV also favors right unusual grafts because it has a separate root of right anterior and posterior PV to the main PV, which ensures a longer root for each PV. A separate hepatic duct of the anterior and posterior sections is also important. Likewise, multifactorial considerations are required to select the optimal graft type.

Herein, the immediate postoperative laboratory findings showed a gradual decrease, which were interpreted as good outcomes (Fig. 3). The surge in alanine aminotransferase levels in recipients Case 1 and Case 6 can be explained by acute rejection, which required steroid pulse therapy (Fig. 3-A). Because the recipient Case 7 underwent small-for-size syndrome, the postoperative levels of ALT, total bilirubin, and INR showed different tendencies than that of the other recipients. The postoperative laboratory results of the donors showed a definite tendency of decrement. The one exception is the surge in alanine aminotransferase levels in donor Case 1, who underwent right posterior sectionectomy of the liver. His postoperative computed tomography finding showed an ischemic change in the transection plane, which might have been the reason for the high alanine aminotransferase level. Besides this, donor Case 1 had no other distinct laboratory findings.

**Fig. 5:**
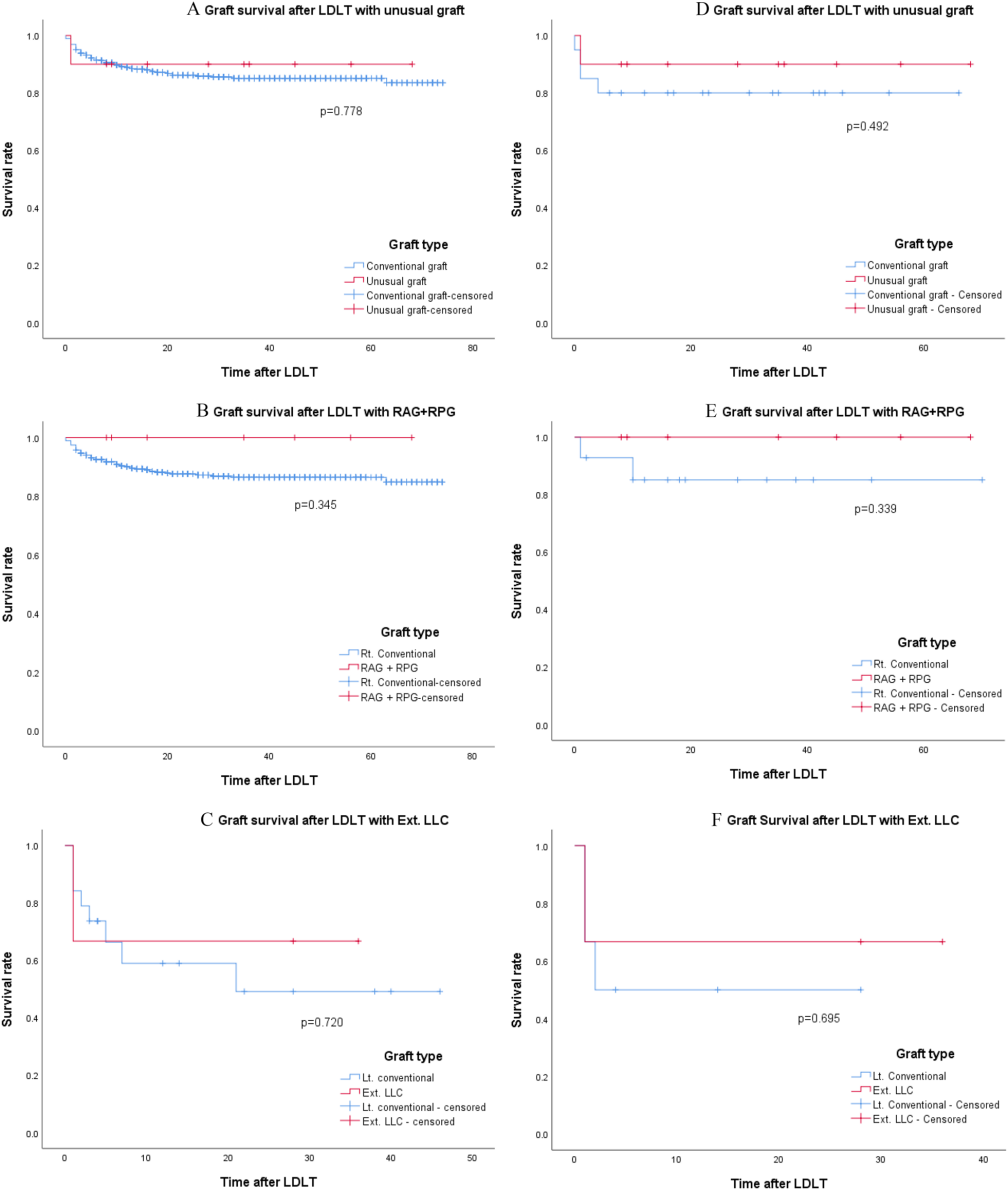
Kaplan–Meier survival curves of patients who had undergone living donor liver transplantation with the unusual graft. Graft survival according to graft types before propensity-score matching (**A**–**C**) and after propensity-score matching (**D**–**F**). Ext. LLC: extended left liver plus caudate lobe graft; LDLT: living-donor liver transplantation; RAG: right anterior section graft; RPG: right posterior section graft

The postoperative computed tomography scans of the recipients revealed intact flow in HA, PV, and HV, which were interpreted as successful outcomes (Fig. 5). However, one complication of PV anastomosis narrowing was observed in Case 4.

The complication rate of the unusual graft group was not significantly different from that of the conventional graft group. A detailed description of the major complication is as follows. Right anterior section graft recipient Case 5 underwent re-do anastomosis of the HA due to HA thrombosis. Additionally, during the follow-up period, percutaneous transhepatic biliary drainage was performed because of the anastomotic biliary stricture. Right posterior section graft recipient Case 2 underwent endoscopic retrograde biliary drainage stent insertion, and Case 8 underwent endoscopic retrograde biliary drainage and percutaneous transhepatic biliary drainage with rendezvous technique due to anastomotic biliary stricture. Case 4, which is the recipient of extended left liver plus caudate lobe graft, underwent PV stent insertion due to the narrowing of PV, and percutaneous transhepatic biliary drainage due to anastomotic biliary stricture. Case 7 suffered small-for-size syndrome. From the perspective of donor safety, unusual grafts did not yield a bad outcome. Only one case (Case 1) showed donor complication (*p* = 0.169), which was a wound seroma, a Clavien–Dindo classification II complication, which occurred on postoperative day 9. No other donor complications were observed in any other case, and the overall complication rate showed no significant difference (*p* = 0.169). Lee et at. reported the complication rates of LDLT donors from The Korean Organ Transplantation Registry (KOTRY) study [21]. In the KOTRY study, the overall complication rate of the donors was 9.3% and the major complication rate was 1.9%. Comparing the complication rate of unusual graft donors in this study to that of the KOTRY group, we can say that the unusual graft shows a safe outcome for donors.

Unusual grafts have several difficulties, and the major issue is the transection plane. For right anterior section graft and posterior section graft, the second-order bifurcation of the right HA needs to be dissected. Not only this procedure faces many variations, but also time-consuming. Especially right anterior section graft has two transection plane which required delicate transection along hepatic veins. Furthermore, the difficult transection plane could lead to the infarction of the liver that affects not only the graft—which results in graft volume loss—but also the residual liver of the donor. It can be a critical weak point of the unusual graft, because small-for-size syndrome is a life-threatening complication for the recipient. Therefore, we should secure sufficient GRWR to avoid the risk of small-for-size syndrome in recipient. In addition to graft volume, reconstruction feasibility is another issue. Because the difficult transection plane leads to multiple vessel stumps or structures that are difficult to make engraftment. Furthermore, the unusual grafts exhibited significantly smaller and fragile stumps compared to conventional graft. As a result, additional reconstruction in bench surgery and time-consuming anastomosis were necessary, leading to prolonged ischemic time. Although no statistical difference was found, a higher incidence of biliary complications was observed in cases involving unusual graft when compared to those with conventional graft.

Despite all these adversities, recent advance in technologies gives us the chance to prepare for these difficulties. Three-dimensional reconstruction of the donor’s liver is getting more and more precise, and volumetry of the graft is becoming more accurate. With this detailed information, the diverse variants of the liver can be more accurately expected, and we can select donors more precisely.

This study has several limitations to consider. First, it is a single-center study with retrospective design. It has potential limited generalizability; however, it also has homogeneity of surgical method, immunosuppressive regimen, and follow-up protocols. Second, small case numbers; we only had two cases of right anterior section graft and three cases of extended left lobe plus caudate lobe graft. Therefore, further studies on unusual grafts for LDLT in a larger number of patients should be conducted.

In conclusion, the results of this study suggest that unusual grafts offer acceptable surgical outcomes. As unusual grafts have been observed to be safe, these grafts can be considered alternatives when conventional grafts cannot be obtained from donors. Considering the shortage of LDLT donors, unusual grafts may extend the donor pool for LDLT with acceptable surgical outcomes.

## Data Availability

The datasets generated and analyzed during the current study are not available to the public due to the violation of patient privacy and the lack of informed consent for online raw data. However, they are available upon reasonable request from the corresponding author in question.

## Abbreviations

GRWR: Graft–recipient weight ratio
HA: Hepatic artery
HD: Hepatic duct
HV: Hepatic vein
INR: International normalized ratio
LDLT: Living-donor liver transplantation
LT: Liver transplantation
MELD: Model for end-stage liver disease
PSM: Propensity score matching
PV: Portal vein
